# Editorial

**Published:** 1875-03

**Authors:** 


### Editorial.

### NEW GOLD.

Dr. C. E. Blake, of San Francisco, recently made us a flying visit when he presented some heavy gold foil, prepared with a view of securing some points not heretofore attained, viz: a gold foil No. 60 the body of which contains twenty per cent of platinum, and this alloy covered with pure gold, the the object of this is to secure a hard gold, for the surface of fillings that are subjected to wear in mastication, the surface consisting of pure gold, makes it very cohesive, and it welds as well as though it was wholly of fine gold. On all gold fillings where there is much wear by mastication it will be of great service. Another foil No. 60 he is introducing which he denominates "Proof Gold" by which he means gold absolutely pure. This he claims has not been attained by any other maker of foil. This foil is very soft and exceedingly cohesive, and is well adapted for making fillings anywhere. These foils are not yet in the market, at least outside of San Francisco; but it is the intention of Dr. Blake to make arrangements very soon to supply the profession, with these preparations of gold; and also some others in which there seems to be some excellent qualities developed.

### THE AMERICAN DENTAL SOCIETY OF EUROPE.

The American Dental Society of Europe will meet at Homburg, near Frankfort, on the Main, on the first Monday in August, 1875.

American brothers who may be traveling in Europe, are invited to arrange their trip, so that we may welcome them in Homburg C. M. Wright Sec.

Basel, Switzerland.

### FLOWERS.

Every dentist should be a lover of the beautiful; and no where in nature can beauty be better and more pleasingly studied than in flowers. The rearing, study and admiration of them, constitutes a grateful relaxation, and diversion from the severe and arduous labors of the dentist in full practice. We have a very forcible reminder in this direction, by looking over the first number of Vick's Quarterly Guide for 1875, the January number has just come to hand, it contains over one hundred pages, and engravings and descriptions of more than five-hundred of our best flowers and vegetables, with directions for culture. This is the best work of the kind we have ever seen and there is perhaps none better in the world. We advise everybody to procure it, and if its contents are examined, it will stimulate a love for flowers, and thereby develop the finer sensibilities, that in too many of us are latent.

This little work of beauty is published by Jas. Vick, Rochester N. Y., at 25 cents a year.

### S. S. WHITE DENTAL ENGINE.

We take pleasure in thus acknowledging the reception, by the Ohio Dental College, of one of Mr. S. S. White's superb Dental Engines. The completeness and perfection of which, leaves little if anything more to be desired. It certainly has the best hand piece, for retaining the drill, and we think in all respects, extant; this will be fully appreciated by those who try it, after having been annoyed with those from which the drill falls whenever they have a downward inclination.

These engines, like everything from Mr. White's establishment, have a beauty and finish, that will be difficult to equal, and more difficult to excel.

The thanks of the faculty are hereby extended to Mr. White for this valuable contribution to the practical department of the Institution.

